# Non-fragile mixed *H*_∞_ and passive synchronization of Markov jump neural networks with mixed time-varying delays and randomly occurring controller gain fluctuation

**DOI:** 10.1371/journal.pone.0175676

**Published:** 2017-04-14

**Authors:** Chao Ma

**Affiliations:** School of Automation and Electrical Engineering, University of Science and Technology Beijing, Beijing 100083, China; Lanzhou University of Technology, CHINA

## Abstract

This paper studies the non-fragile mixed *H*_∞_ and passive synchronization problem for Markov jump neural networks. The randomly occurring controller gain fluctuation phenomenon is investigated for non-fragile strategy. Moreover, the mixed time-varying delays composed of discrete and distributed delays are considered. By employing stochastic stability theory, synchronization criteria are developed for the Markov jump neural networks. On the basis of the derived criteria, the non-fragile synchronization controller is designed. Finally, an illustrative example is presented to demonstrate the validity of the control approach.

## Introduction

There have been significant attentions on dynamic behaviors of neural networks, since they have various current and future potential applications, i.e., signal processing, optimization problems, pattern recognition and so forth. [1–9]. In particular, the study of Markov jump neural networks has been a significant topic during the past years, since this model can better describe the neural networks with different structures in real life. Generally speaking, the mode jumps displayed in the Markov jump neural networks are commonly considered to be governed by an ideal homogeneous Markov chain. With the help of analysis and synthesis for Markov jump systems, some remarkable results on Markov jump neural networks have been reported in the literature and the references therein [10–19].

On another research line, the synchronization problem has become a hot topic in the fields of neural networks [9, 12]. When one neural network can synchronize the other neural network, they can display complicated dynamic behaviors, which can give an insight into the characteristics of neural network. As is well known, time delays exist in neural networks, such that there is a need for the synchronization problem with time delays [20, 21]. Moreover, it is noted that another unavoidable factor affecting the synchronization in neural networks is the disturbance. As a result, several effective synchronization strategies for neural networks with disturbances have been proposed, especially for some finite-time cases [22–28].

It is worth mentioning that the theory of passivity has provided a powerful tool in analyzing and synthesis of complex dynamic systems [29, 30]. Note that some initial researches are on the mixed *H*_∞_ and passive filtering design, which can provide a more flexible design than common *H*_∞_ approach [31]. In addition, the non-fragile synchronization controller should be designed for controller gain fluctuation attenuation [32]. Furthermore, it can be found that the controller gain fluctuation can happen in a stochastic way [33]. Consequently, a natural question arises: how to solve the synchronization problem for Markov jump neural networks? Unfortunately, up to now, such a question has not been fully addressed and remains open.

This paper is to deal with the above question. In this paper, a stochastic variable is adopted for describing the controller gain fluctuation. Based on stochastic methods, synchronization criteria are first established, such that the drive and response Markov jump neural networks can be synchronized in the presence of mixed time-varying delays and disturbance. Base on the derived results, a design procedure is given for the synchronization controller.

The remainder of the paper is arranged as follows. The Markov jump neural network model is first introduced, and the non-fragile synchronization problem is formulated. The main results of the synchronization problem are then provided. Moreover, the simulation results are given and this paper is concluded in the end.

**Notation**: $R^{n}$ denotes the *n* dimensional Euclidean space, $R^{m\times n}$ denotes the set of *m* × *n* matrices. $L_{2}\left[0,\infty \right)$ denotes the space of square-integrable vector functions over [0, ∞). (Ω,F,P) is a probability space, Ω is the sample space, F is the *σ*-algebra of subsets of the sample space and P is the probability measure on F. Pr{*α*} means the occurrence probability of the event *α*, and Pr{*α*|*β*} means the occurrence probability of *α* conditional on *β*. E{x} means the expectation of the stochastic variable *x* and E{x|y} means the expectation of the stochastic variable *x* conditional on the stochastic variable *y*. * denotes the ellipsis in symmetric block matrices and diag{⋯} denotes a block-diagonal matrix.

## Methods

Consider the Markov jump neural networks with mixed time-varying delays in (Ω,F,P):

$$\begin{array}{ccc} \dot{x}\left(t\right) & = & -C(r(t))x(t)+A(r(t))f(x(t))+B(r(t))f(x(t-\tau (t)))\\ & & +D\left(r\left(t\right)\right)\int_{t-d(t)}^{t}f\left(x\left(s\right)\right)ds \end{array}$$ (1)

where *x*(*t*) = [*x*_1_(*t*), *x*_2_(*t*), …, *x*_*n*_(*t*)]^*T*^ denotes the state of the neuron; *f*(*x*(*t*)) = [*f*_1_(*x*_1_(*t*)), *f*_2_(*x*_2_(*t*)), …, *f*_*n*_(*x*_*n*_(*t*))]^*T*^ is the neuron activation function; $C\left(r\left(t\right)\right)=\text{diag}\{c_{1}\left(r\left(t\right)\right),c_{2}\left(r\left(t\right)\right),\ldots ,c_{n}\left(r\left(t\right)\right)\}$ is a diagonal matrix with positive entries; Matrices *A*(*r*(*t*)) = (*a*_*ij*_(*r*(*t*)))_*n*×*n*_, *B*(*r*(*t*)) = (*b*_*ij*_(*r*(*t*)))_*n*×*n*_ and *D*(*r*(*t*)) = (*d*_*ij*_(*r*(*t*)))_*n*×*n*_ represent the connection weight matrix, the discretely delayed connection weight matrix and the distributively delayed connection weight matrix, respectively; *τ*(*t*) and *d*(*t*) denote the discrete delay and distributed delay, respectively, which satisfy

$$\begin{array}{ccc} 0 & \leq & \tau \left(t\right)\leq \bar{\tau },\dot{\tau }\left(t\right)\leq \mu <1, \end{array}$$ (2)

$$\begin{array}{ccc} 0 & \leq & d\left(t\right)\leq \bar{d}, \end{array}$$ (3)

where $\bar{\tau }$, *μ*, and $\bar{d}$ are known positive constants. The initial condition of Eq (1) is given by *x*(*s*) = *ϕ*(*s*), $s\in [-\max\left\{\bar{\tau },\bar{d}\right\},0]$.

{*r*(*t*), *t* ≥ 0} is a right continuous continuous-time Markov process with $\Theta =\{\pi_{ij}\},\forall i,j\in I$ described as

$$\begin{array}{c} \mathrm{Pr}\left(r\left(t+\Delta t\right)=j:r\left(t\right)=i\right)=\{\begin{array}{c} \pi_{ij}\Delta t+o\left(\Delta t\right)\;\;\;\;\;\;\;\;\;\text{if}\;i\neq j\\ 1+\pi_{ii}\Delta t+o\left(\Delta t\right)\;\;\;\;\;\text{if}\;i=j \end{array} \end{array}$$ (4)

with Δ*t* > 0, lim(*o*(Δ*t*)/Δ*t*) = 0 and *π*_*ij*_ ≥ 0 (i,j∈I,j≠i) is the transition rate from mode *i* at time *t* to mode *j* at time *t* + Δ*t*, while $\pi_{ii}=-\sum_{j=1,\;j\neq i}^{N}\pi_{ij\;\;}$ for ∀i∈I.

**Assumption 1.** *The activation function* *f*(*x*(*t*)) *in* Eq (1) *is continuous and bounded, and satisfies*

$$\begin{array}{c} F_{i}^{-}\leq \frac{f_{i}\left(\alpha \right)-f_{i}\left(\beta \right)}{\alpha -\beta }\leq F_{i}^{+},i=1,2,\ldots ,n, \end{array}$$ (5)

*where* *f*_*i*_(0) = 0, *α*, β∈R, *α* ≠ *β* *and* $F_{i}^{-}$ *and* $F_{i}^{+}$ *are known real constants*.

Denote Eq (1) as the drive neural network. For the sake of simplicity, we denote the Markov process *r*(*t*) by *i* indices. Moreover, it is assumed that the mode of the drive and the response neural networks can be identical all the time [34]. Then, the response neural network can be given by

$$\begin{array}{ccc} \dot{y}\left(t\right) & = & -C_{i}y\left(t\right)+A_{i}f\left(y\left(t\right)\right)+B_{i}f\left(y\left(t-\tau \left(t\right)\right)\right)\\ & & +D_{i}\int_{t-d(t)}^{t}f\left(y\left(s\right)\right)ds+u\left(t\right)+\varpi \left(t\right), \end{array}$$ (6)

where *u*(*t*) denotes the control input and $\varpi \left(t\right)\in L_{2}\left[0,\infty \right)$ is the disturbance.

Define synchronization error as

$$\begin{array}{c} e(t)=y(t)-x(t), \end{array}$$ (7)

then it follows that

$$\begin{array}{ccc} \dot{e}\left(t\right) & = & -C_{i}e\left(t\right)+A_{i}\left(f\left(y\left(t\right)\right)-f\left(x\left(t\right)\right)\right)\\ & & +B_{i}\left(f\left(y\left(t-\tau \left(t\right)\right)\right)-f\left(x\left(t-\tau \left(t\right)\right)\right)\right)\\ & & +D_{i}\int_{t-d(t)}^{t}\left(f\left(y\left(s\right)\right)-f\left(x\left(s\right)\right)\right)ds\\ & & +u(t)+\varpi (t). \end{array}$$ (8)

We develop the following mode-dependent controller as:

$$\begin{array}{c} u\left(t\right)=(K_{i}+\delta \left(t\right)\Delta K_{i})e\left(t\right), \end{array}$$ (9)

where $K_{i}\in R^{n\times n}$ is the mode-dependent controller gain and Δ*K*_*i*_ is the controller gain fluctuation with

$$\begin{array}{c} \Delta K_{i}=H_{i}{ϒ}_{i}\left(t\right)E_{i}, \end{array}$$ (10)

where *H*_*i*_ and *E*_*i*_ are known constant matrices, ${ϒ}_{i}\left(t\right)\in R^{k\times l}$ satisfies

$$\begin{array}{c} {ϒ}_{i}^{T}\left(t\right){ϒ}_{i}\left(t\right)\leq I_{k}. \end{array}$$ (11)

δ(t)∈R is defined by

$$\begin{array}{c} \delta \left(t\right)=\{\begin{array}{cc} 1, & \;\text{controller}\;\text{gain}\;\text{fluctuation}\;\text{happens,}\\ 0, & \;\text{controller}\;\text{gain}\;\text{fluctuation}\;\text{does}\;\text{not}\;\text{happen,} \end{array} \end{array}$$ (12)

with

$$\begin{array}{ccc} \mathrm{Pr}\{\delta (t)=1\} & = & \delta , \end{array}$$ (13)

$$\begin{array}{ccc} \mathrm{Pr}\{\delta (t)=0\} & = & 1-\delta , \end{array}$$ (14)

where *δ* ∈ [0, 1] is a known constant.

Consequently, System (8) can be rewritten as

$$\begin{array}{ccc} \dot{e}\left(t\right) & = & \left(K_{i}+\delta \left(t\right)\Delta K_{i}-C_{i}\right)e\left(t\right)+A_{i}\left(f\left(y\left(t\right)\right)-f\left(x\left(t\right)\right)\right)\\ & & +B_{i}\left(f\left(y\left(t-\tau \left(t\right)\right)\right)-f\left(x\left(t-\tau \left(t\right)\right)\right)\right)\\ & & +D_{i}\int_{t-d(t)}^{t}\left(f\left(y\left(s\right)\right)-f\left(x\left(s\right)\right)\right)ds+\varpi \left(t\right). \end{array}$$ (15)

The following definitions and lemmas are introduced.

**Definition 1.** [31] System (15) *is said to have mixed* *H*_∞_ *and passive performance* *γ*, *if there exists a constant* *γ* > 0 *such that*

$$\begin{array}{ccc} & & \int_{0}^{t_{p}}\left(-\gamma^{-1}\theta e^{T}\left(s\right)e\left(s\right)+2\left(1-\theta \right)e^{T}\left(s\right)\varpi \left(s\right)\right)ds\\ & & \;\geq -\gamma \int_{0}^{t_{p}}\varpi^{T}\left(s\right)\varpi \left(s\right)ds, \end{array}$$ (16)

*for all* *t*_*p*_ > 0 *and any non-zero* $\varpi \left(t\right)\in L_{2},$ *where* *θ* ∈ [0, 1] *represents the parameter that defines the trade-off between* *H*_∞_ *and passive performance*.

**Definition 2.** *The mixed* *H*_∞_ *and passive synchronization of the Markov jump neural networks* Eqs (1) *and* (6) *is said to be achieved if* System (15) *can achieve the mixed* *H*_∞_ *and passive performance with the prescribed* *γ*.

**Lemma 1.** [35] *For any positive symmetric constant matrix* $Q\in R^{n\times n}$, *scalars* *h*_1_, *h*_2_ *satisfying* *h*_1_ < *h*_2_, *a vector function* $\phi :\left[h_{1},h_{2}\right]\to R^{n}$, *such that the integrations concerned are well defined, then*

$$\begin{array}{ccc} & & {\left(\int_{h_{1}}^{h_{2}}\phi \left(s\right)ds\right)}^{T}Q(\int_{h_{1}}^{h_{2}}\phi \left(s\right)ds)\\ & & \;\leq \left(h_{2}-h_{1}\right){\left(\int_{h_{1}}^{h_{2}}\phi^{T}\left(s\right)Q\phi \left(s\right)ds\right)}^{T}. \end{array}$$ (17)

**Lemma 2.** [36] *For any matrix* *M* > 0, *scalars* *τ* > 0, *τ*(*t*) *satisfying* 0 ≤ *τ*(*t*)≤*τ*, *vector function* $\dot{x}\left(t\right):\left[-\tau ,0\right]\to R^{n}$ *such that the concerned integrations are well defined, then*

$$\begin{array}{c} -\tau \int_{t-\tau }^{t}{\dot{x}}^{T}\left(s\right)M\dot{x}\left(s\right)ds\leq \zeta^{T}\left(t\right)\Omega \zeta \left(t\right), \end{array}$$ (18)

*where*

$$\begin{array}{ccc} \zeta (t) & = & {\left[x^{T}\left(t\right),x^{T}\left(t-\tau \left(t\right)\right),x^{T}\left(t-\tau \right)\right]}^{T}, \end{array}$$ (19)

$$\begin{array}{ccc} \Omega & = & [\begin{array}{ccc} -M & M & 0\\ {}* & -2M & M\\ {}* & * & -M \end{array}]. \end{array}$$ (20)

**Lemma 3.** [37] *Let* *L*^*T*^ = *L*, *H* *and* *E* *be real matrices of appropriate dimensions with* *F*(*t*) *satisfying* *F*^*T*^(*t*)*F*(*t*) ≤ *I*. *Then*, *L* + *HFE* + *E*^*T*^ *F*^*T*^ *H*^*T*^ < 0, *if and only if there exists a scalar* *ε* > 0 *such that* *L* + *ε*^−1^ *HH*^*T*^ + *εE*^*T*^ *E* < 0, *or equivalently*

$$\begin{array}{c} {}[\begin{array}{ccc} L & H & \varepsilon E^{T}\\ {}* & -\varepsilon I & 0\\ {}* & * & -\varepsilon I \end{array}]<0. \end{array}$$ (21)

## Results

In this section, delay-dependent synchronization conditions will be developed, based on which the non-fragile synchronization controller is designed.

**Theorem 1.** *For given upper bounds of mixed time-varying delays* $\bar{\tau }$ *and* $\bar{d}$, *and given scalars* *θ* *and* *γ*, *the mixed* *H*_∞_ *and passive synchronization of the Markov jump neural networks* Eqs (1) *and* (6) *can be achieved in the sense of Definition 1 and 2, if there exist mode-dependent symmetric matrices* *P*_*i*_ > 0, *symmetric matrices* *Q* > 0, *R* > 0 *and a constant* *ε* > 0 *such that*

$$\begin{array}{c} \Pi_{i}:=[\begin{array}{cc} \Pi_{i1} & \Pi_{i2}\\ {}* & \Pi_{i3} \end{array}]<0 \end{array}$$ (22)

*hold for all* i∈I, *respectively, where*

$$\begin{array}{ccc} \hat{F} & := & \mathrm{diag}\{F_{1}^{-}F_{1}^{+},F_{2}^{-}F_{2}^{+},\ldots ,F_{n}^{-}F_{n}^{+}\}, \end{array}$$ (23)

$$\begin{array}{ccc} \overset{ˇ}{F} & := & \mathrm{diag}\{\frac{F_{1}^{-}+F_{1}^{+}}{2},\frac{F_{2}^{-}+F_{2}^{+}}{2},\ldots ,\frac{F_{n}^{-}+F_{n}^{+}}{2}\}, \end{array}$$ (24)

$$\begin{array}{ccc} \Pi_{i1} & := & [\begin{array}{cc} \Pi_{i11} & \Pi_{i12}\\ {}* & \Pi_{i13} \end{array}], \end{array}$$ (25)

$$\begin{array}{ccc} \Pi_{i11} & := & [\begin{array}{cc} \begin{array}{c} P_{i}K_{i}+K_{i}^{T}P_{i}^{T}-P_{i}C_{i}-C_{i}^{T}P_{i}^{T}+\underset{j=1}{\sum^{N}}\pi_{ij}P_{j}\\ +Q-R-\hat{F}\Lambda_{1i}+\gamma^{-1}\theta I \end{array} & R\\ {}* & -2R-\hat{F}\Lambda_{2i} \end{array}], \end{array}$$ (26)

$$\begin{array}{ccc} \Pi_{i12} & := & [\begin{array}{ccc} 0 & P_{i}-\left(1-\theta \right)I & P_{i}A_{i}+\overset{ˇ}{F}\Lambda_{1i}\\ R & 0 & \overset{ˇ}{F}\Lambda_{2i} \end{array}], \end{array}$$ (27)

$$\begin{array}{ccc} \Pi_{i13} & := & \mathrm{diag}\{-Q-R,-\gamma I,-\Lambda_{1i}+{\bar{d}}^{2}W\}, \end{array}$$ (28)

$$\begin{array}{ccc} \Pi_{i2} & := & [\begin{array}{cccccc} P_{i}B_{i} & P_{i}D_{i} & \bar{\tau }K_{i}^{T}P_{i}-\bar{\tau }C_{i}^{T}P_{i} & 0 & \delta P_{i}H_{i} & \varepsilon E_{i}^{T}\\ 0 & 0 & 0 & 0 & 0 & 0\\ 0 & 0 & 0 & 0 & 0 & 0\\ 0 & 0 & \bar{\tau }P_{i} & 0 & 0 & 0\\ 0 & 0 & \bar{\tau }A_{i}^{T}P_{i} & 0 & 0 & 0 \end{array}], \end{array}$$ (29)

$$\begin{array}{ccc} \Pi_{i3} & := & [\begin{array}{cccccc} -\Lambda_{2i} & 0 & \bar{\tau }B_{i}^{T}P_{i} & 0 & 0 & 0\\ {}* & -W & \bar{\tau }D_{i}^{T}P_{i} & 0 & 0 & 0\\ {}* & * & R-2P_{i} & 0 & \bar{\tau }\delta P_{i}H_{i} & 0\\ {}* & * & * & R-2P_{i} & \sqrt{\delta (1-\delta )}\bar{\tau }P_{i}C_{i}H_{i} & 0\\ {}* & * & * & * & -\varepsilon I & 0\\ {}* & * & * & * & * & -\varepsilon I \end{array}]. \end{array}$$ (30)

*proof.* Choose the Lyapunov-Krasovskii function as:

$$\begin{array}{c} V\left(t\right)=\sum_{i=1}^{4}V_{i}\left(t\right), \end{array}$$ (31)

where

$$\begin{array}{ccc} V_{1}\left(t\right) & := & e^{T}\left(t\right)P_{i}e\left(t\right), \end{array}$$ (32)

$$\begin{array}{ccc} V_{2}\left(t\right) & := & \int_{t-\bar{\tau }}^{t}e^{T}\left(\varphi \right)Qe\left(\varphi \right)d\varphi , \end{array}$$ (33)

$$\begin{array}{ccc} V_{3}\left(t\right) & := & \bar{\tau }\int_{-\bar{\tau }}^{0}\int_{t+\eta }^{t}{\dot{e}}^{T}\left(\varphi \right)R\dot{e}\left(\varphi \right)d\varphi d\eta , \end{array}$$ (34)

$$\begin{array}{ccc} V_{4}\left(t\right) & := & \bar{d}\int_{-\bar{d}}^{0}\int_{t+\eta }^{t}{\tilde{f}}^{T}\left(e\left(\varphi \right)\right)W\tilde{f}\left(e\left(\varphi \right)\right)d\varphi d\eta , \end{array}$$ (35)

$$\begin{array}{ccc} \tilde{f}\left(e\left(t\right)\right) & := & f(y(t))-f(x(t)). \end{array}$$ (36)

The infinitesimal operator L of *V*(*t*) is defined by

$$\begin{array}{c} LV\left(t\right)=\lim_{\Delta \to 0^{+}}\frac{1}{\Delta }\left\{E\left\{V\left(t+\Delta \right)|t\right\}-V\left(t\right)\right\}. \end{array}$$ (37)

Then for each mode *i*, by taking the derivative of Eq (31) along the solution of System (15), one has

$$\begin{array}{ccc} E\{LV_{1}\left(t\right)\} & = & E\{e^{T}\left(t\right)P_{i}\dot{e}\left(t\right)+{\dot{e}}^{T}\left(t\right)P_{i}e\left(t\right)+\sum_{j=1}^{N}\pi_{ij}e^{T}\left(t\right)P_{j}e\left(t\right)\},\\ & = & E\{2e^{T}\left(t\right)P_{i}(\left(K_{i}+\delta \left(t\right)\Delta K_{i}-C_{i}\right)e\left(t\right)+A_{i}\left(f\left(y\left(t\right)\right)-f\left(x\left(t\right)\right)\right)\\ & & +B_{i}\left(f\left(y\left(t-\tau \left(t\right)\right)\right)-f\left(x\left(t-\tau \left(t\right)\right)\right)\right)+D_{i}\int_{t-d(t)}^{t}(f\left(y\left(\varphi \right)\right)\\ & & -f\left(x\left(\varphi \right)\right))d\varphi +\varpi \left(t\right))+\sum_{j=1}^{N}\pi_{ij}e^{T}\left(t\right)P_{j}e\left(t\right)\}\\ & = & E\{2e^{T}\left(t\right)P_{i}(\left(K_{i}+\delta \Delta K_{i}-C_{i}\right)e\left(t\right)+A_{i}\tilde{f}\left(e\left(t\right)\right)\\ & & +B_{i}\tilde{f}\left(e\left(t-\tau \left(t\right)\right)\right)+D_{i}\int_{t-d(t)}^{t}\tilde{f}\left(e\left(\varphi \right)\right)d\varphi \\ & & +\varpi \left(t\right))+\sum_{j=1}^{N}\pi_{ij}e^{T}\left(t\right)P_{j}e\left(t\right)\}, \end{array}$$ (38)

$$\begin{array}{ccc} E\{LV_{2}\left(t\right)\} & = & E\{e^{T}\left(t\right)Qe\left(t\right)-e^{T}\left(t-\bar{\tau }\right)Qe\left(t-\bar{\tau }\right)\}, \end{array}$$ (39)

$$\begin{array}{ccc} E\{LV_{3}\left(t\right)\} & = & E\{{\bar{\tau }}^{2}{\dot{e}}^{T}\left(t\right)R\dot{e}\left(t\right)-\bar{\tau }\int_{t-\bar{\tau }}^{t}{\dot{e}}^{T}\left(\varphi \right)R\dot{e}\left(\varphi \right)d\varphi \}, \end{array}$$ (40)

$$\begin{array}{ccc} E\{LV_{4}\left(t\right)\} & = & E\{{\bar{d}}^{2}{\tilde{f}}^{T}\left(e\left(t\right)\right)W\tilde{f}\left(e\left(t\right)\right)-\bar{d}\int_{t-\bar{d}}^{t}{\tilde{f}}^{T}\left(e\left(\varphi \right)\right)W\tilde{f}\left(e\left(\varphi \right)\right)d\varphi \}. \end{array}$$ (41)

By Lemma 1 and Lemma 2, it holds that:

$$\begin{array}{ccc} & & -\bar{d}\int_{t-\bar{d}}^{t}{\tilde{f}}^{T}\left(e\left(\varphi \right)\right)W\tilde{f}\left(e\left(\varphi \right)\right)d\varphi \\ & & \;\leq -\int_{t-\bar{d}}^{t}{\tilde{f}}^{T}\left(e\left(\varphi \right)\right)W\int_{t-\bar{d}}^{t}\tilde{f}\left(e\left(\varphi \right)\right)d\varphi \\ & & \leq -\int_{t-d(t)}^{t}{\tilde{f}}^{T}\left(e\left(\varphi \right)\right)d\varphi W\int_{t-d(t)}^{t}\tilde{f}\left(e\left(\varphi \right)\right)d\varphi , \end{array}$$ (42)

$$\begin{array}{ccc} & & -\bar{\tau }\int_{t-\bar{\tau }}^{t}{\dot{e}}^{T}\left(\varphi \right)R\dot{e}\left(\varphi \right)d\varphi \\ & & \leq {\left[\begin{array}{c} x(t)\\ x(t-\tau (t))\\ x(t-\bar{\tau }) \end{array}\right]}^{T}[\begin{array}{ccc} -R & R & 0\\ {}* & -2R & R\\ {}* & * & -R \end{array}]\times \\ & & \left[\begin{array}{c} x(t)\\ x(t-\tau (t))\\ x(t-\bar{\tau }) \end{array}\right]. \end{array}$$ (43)

It follows from Assumption 1 that

$$\begin{array}{ccc} F_{i}^{-} & \leq & \frac{f_{i}\left(y\left(t\right)\right)-f_{i}\left(x\left(t\right)\right)}{e(t)}\leq F_{i}^{+}, \end{array}$$ (44)

$$\begin{array}{ccc} F_{i}^{-} & \leq & \frac{f_{i}\left(y\left(t-\tau \left(t\right)\right)\right)-f_{i}\left(x\left(t-\tau \left(t\right)\right)\right)}{e(t-\tau (t))}\leq F_{i}^{+}, \end{array}$$ (45)

such that the following inequality holds

$$\begin{array}{ccc} {\left[\begin{array}{c} e(t)\\ \tilde{f}\left(e\left(t\right)\right) \end{array}\right]}^{T}[\begin{array}{cc} \hat{F}\Lambda_{1i} & -\overset{ˇ}{F}\Lambda_{1i}\\ {}* & \Lambda_{1i} \end{array}][\begin{array}{c} e(t)\\ \tilde{f}\left(e\left(t\right)\right) \end{array}] & \leq & 0, \end{array}$$ (46)

$$\begin{array}{ccc} {\left[\begin{array}{c} e(t-\tau (t))\\ \tilde{f}\left(e\left(t-\tau \left(t\right)\right)\right) \end{array}\right]}^{T}[\begin{array}{cc} \hat{F}\Lambda_{2i} & -\overset{ˇ}{F}\Lambda_{2i}\\ {}* & \Lambda_{2i} \end{array}][\begin{array}{c} e(t-\tau (t))\\ \tilde{f}\left(e\left(t-\tau \left(t\right)\right)\right) \end{array}] & \leq & 0. \end{array}$$ (47)

Define

$$\begin{array}{c} J=\int_{0}^{t_{p}}\left[\gamma^{-1}\theta e^{T}\left(s\right)e\left(s\right)-2\left(1-\theta \right)e^{T}\left(s\right)\varpi \left(s\right)-\gamma \varpi^{T}\left(s\right)\varpi \left(s\right)\right]ds. \end{array}$$ (48)

Noting that $E\left\{{\left(\delta (t)-\delta \right)}^{2}\right\}=\delta \left(1-\delta \right)$, it can be deduced that

$$\begin{array}{ccc} & & E\{LV\left(t\right)+\gamma^{-1}\theta e^{T}\left(s\right)e\left(s\right)-2\left(1-\theta \right)e^{T}\left(s\right)\varpi \left(s\right)-\gamma \varpi^{T}\left(s\right)\varpi \left(s\right)\}\\ & & \;=E\{\sum_{i=1}^{4}V_{i}\left(t\right)+\gamma^{-1}\theta e^{T}\left(s\right)e\left(s\right)-2\left(1-\theta \right)e^{T}\left(s\right)\varpi \left(s\right)-\gamma \varpi^{T}\left(s\right)\varpi \left(s\right)\}\\ & & \;\leq \eta^{T}\left(t\right){\bar{\Pi }}_{i}\eta \left(t\right), \end{array}$$ (49)

where

$$\begin{array}{ccc} {\bar{\Pi }}_{i} & = & {\tilde{\Pi }}_{i}+[\begin{array}{c} \bar{\tau }{\left(K_{i}+\delta \Delta K_{i}-C_{i}\right)}^{T}\\ 0\\ 0\\ \bar{\tau }I\\ \bar{\tau }A_{i}^{T}\\ \bar{\tau }B_{i}^{T}\\ \bar{\tau }D_{i}^{T} \end{array}]R{\left[\begin{array}{c} \bar{\tau }{\left(K_{i}+\delta \Delta K_{i}-C_{i}\right)}^{T}\\ 0\\ 0\\ \bar{\tau }I\\ \bar{\tau }A_{i}^{T}\\ \bar{\tau }B_{i}^{T}\\ \bar{\tau }D_{i}^{T} \end{array}\right]}^{T}\\ & & +[\begin{array}{c} \sqrt{\delta (1-\delta )}\bar{\tau }\Delta K_{i}^{T}C_{i}^{T}\\ 0\\ 0\\ 0\\ 0\\ 0\\ 0 \end{array}]R{\left[\begin{array}{c} \sqrt{\delta (1-\delta )}\bar{\tau }\Delta K_{i}^{T}C_{i}^{T}\\ 0\\ 0\\ 0\\ 0\\ 0\\ 0 \end{array}\right]}^{T}, \end{array}$$ (50)

$$\begin{array}{ccc} {\tilde{\Pi }}_{i} & = & [\begin{array}{cc} {\tilde{\Pi }}_{i1} & {\tilde{\Pi }}_{i2}\\ {}* & {\tilde{\Pi }}_{i3} \end{array}], \end{array}$$ (51)

$$\begin{array}{ccc} {\tilde{\Pi }}_{i1} & = & [\begin{array}{cc} \begin{array}{c} 2P_{i}\left(K_{i}+\delta \Delta K_{i}-C_{i}\right)+\sum_{j=1}^{N}\pi_{ij}P_{j}\\ +Q-R-\hat{F}\Lambda_{1i}+\gamma^{-1}\theta I \end{array} & R\\ {}* & -2R-\hat{F}\Lambda_{2i} \end{array}], \end{array}$$ (52)

$$\begin{array}{ccc} {\tilde{\Pi }}_{i2} & = & [\begin{array}{ccccc} 0 & P_{i}-\left(1-\theta \right)I & P_{i}A_{i}+\overset{ˇ}{F}\Lambda_{1i} & P_{i}B_{i} & P_{i}D_{i}\\ R & 0 & \overset{ˇ}{F}\Lambda_{2i} & 0 & 0 \end{array}], \end{array}$$ (53)

$$\begin{array}{ccc} {\tilde{\Pi }}_{i3} & = & \text{diag}\{-Q-R,-\gamma I,-\Lambda_{1i}+{\bar{d}}^{2}W,-\Lambda_{2i},-W\}, \end{array}$$ (54)

$$\begin{array}{ccc} \eta (t) & = & {\left[\eta_{1}^{T}\left(t\right),\eta_{2}^{T}\left(t\right)\right]}^{T}, \end{array}$$ (55)

$$\begin{array}{ccc} \eta_{1}\left(t\right) & = & {\left[e^{T}\left(t\right),e^{T}\left(t-\tau \left(t\right)\right),e^{T}\left(t-\bar{\tau }\right),\varpi^{T}\left(t\right)\right]}^{T}, \end{array}$$ (56)

$$\begin{array}{ccc} \eta_{2}\left(t\right) & = & {\left[{\tilde{f}}^{T}\left(e\left(t\right)\right),{\tilde{f}}^{T}\left(e\left(t-\tau \left(t\right)\right)\right),\int_{t-d(t)}^{t}{\tilde{f}}^{T}\left(e\left(\varphi \right)\right)d\varphi \right]}^{T}. \end{array}$$ (57)

By Schur complement [38], it can be obtained that ${\bar{\Pi }}_{i}<0$ is equivalent to ${\hat{\Pi }}_{i}<0$, where

$$\begin{array}{ccc} {\hat{\Pi }}_{i} & = & [\begin{array}{cc} {\hat{\Pi }}_{i1} & {\hat{\Pi }}_{i2}\\ {}* & {\hat{\Pi }}_{i3} \end{array}], \end{array}$$ (58)

$$\begin{array}{ccc} {\hat{\Pi }}_{i1} & = & [\begin{array}{cccc} \begin{array}{c} 2P_{i}\left(K_{i}+\delta \Delta K_{i}-C_{i}\right)+\sum_{j=1}^{N}\pi_{ij}P_{j}\\ +Q-R-\hat{F}\Lambda_{1i}+\gamma^{-1}\theta I \end{array} & R & 0 & P_{i}-\left(1-\theta \right)I\\ {}* & -2R-\hat{F}\Lambda_{2i} & R & 0\\ {}* & * & -Q-R & 0\\ {}* & * & * & -\gamma I \end{array}], \end{array}$$ (59)

$$\begin{array}{ccc} {\hat{\Pi }}_{i2} & = & [\begin{array}{ccccc} P_{i}A_{i}+\overset{ˇ}{F}\Lambda_{1i} & P_{i}B_{i} & P_{i}D_{i} & \bar{\tau }{\left(K_{i}+\delta \Delta K_{i}-C_{i}\right)}^{T} & \sqrt{\delta (1-\delta )}\bar{\tau }\Delta K_{i}{}^{T}C_{i}{}^{T}\\ \overset{ˇ}{F}\Lambda_{2i} & 0 & 0 & 0 & 0\\ 0 & 0 & 0 & 0 & 0\\ 0 & 0 & 0 & \bar{\tau }I & 0 \end{array}], \end{array}$$ (60)

$$\begin{array}{ccc} {\hat{\Pi }}_{i3} & = & [\begin{array}{ccccc} -\Lambda_{1i}+{\bar{d}}^{2}W & 0 & 0 & \bar{\tau }A_{i}^{T} & 0\\ {}* & -\Lambda_{2i} & 0 & \bar{\tau }B_{i}^{T} & 0\\ {}* & * & -W & \bar{\tau }D_{i}^{T} & 0\\ {}* & * & * & -R^{-1} & 0\\ {}* & * & * & * & -R^{-1} \end{array}]. \end{array}$$ (61)

By performing congruence transformation of $\text{diag}\{I,I,I,I,I,I,I,P_{i},P_{i}\}$ to Eq (58) and considering the inequality −*P*_*i*_ *R*^−1^ *P*_*i*_ ≤ *R* − 2*P*_*i*_, ${\hat{\Pi }}_{i}$ can be further rewritten as

$$\begin{array}{ccc} & & {\overset{˘}{\Pi }}_{i}+[\begin{array}{c} \delta P_{i}H_{i}\\ 0\\ 0\\ 0\\ 0\\ 0\\ 0\\ \bar{\tau }\delta P_{i}H_{i}\\ \sqrt{\delta (1-\delta )}\bar{\tau }P_{i}C_{i}H_{i} \end{array}]{ϒ}_{i}\left(t\right)[\begin{array}{ccccccccc} E_{i} & 0 & 0 & 0 & 0 & 0 & 0 & 0 & 0 \end{array}]\\ & & \;+{\left([\begin{array}{c} \delta P_{i}H_{i}\\ 0\\ 0\\ 0\\ 0\\ 0\\ 0\\ \bar{\tau }\delta P_{i}H_{i}\\ \sqrt{\delta (1-\delta )}\bar{\tau }P_{i}C_{i}H_{i} \end{array}]{ϒ}_{i}\left(t\right)[\begin{array}{ccccccccc} E_{i} & 0 & 0 & 0 & 0 & 0 & 0 & 0 & 0 \end{array}]\right)}^{T}, \end{array}$$ (62)

where

$$\begin{array}{ccc} {\overset{˘}{\Pi }}_{i} & = & [\begin{array}{cc} {\overset{˘}{\Pi }}_{i1} & {\overset{˘}{\Pi }}_{i2}\\ {}* & {\overset{˘}{\Pi }}_{i3} \end{array}], \end{array}$$ (63)

$$\begin{array}{c} {\overset{˘}{\Pi }}_{i1}=[\begin{array}{ccc} \begin{array}{c} 2P_{i}\left(K_{i}-C_{i}\right)+\sum_{j=1}^{N}\pi_{ij}P_{j}\\ +Q-R-\hat{F}\Lambda_{1i}+\gamma^{-1}\theta I \end{array} & R & 0\\ {}* & -2R-\hat{F}\Lambda_{2i} & R\\ {}* & * & -Q-R \end{array}], \end{array}$$ (64)

$$\begin{array}{ccc} {\overset{˘}{\Pi }}_{i2} & = & [\begin{array}{cccccc} P_{i}-\left(1-\theta \right)I & P_{i}A_{i}+\overset{ˇ}{F}\Lambda_{1i} & P_{i}B_{i} & P_{i}D_{i} & \bar{\tau }K_{i}^{T}P_{i}-\bar{\tau }C_{i}^{T}P_{i} & 0\\ 0 & \overset{ˇ}{F}\Lambda_{2i} & 0 & 0 & 0 & 0\\ 0 & 0 & 0 & 0 & 0 & 0 \end{array}], \end{array}$$ (65)

$$\begin{array}{ccc} {\overset{˘}{\Pi }}_{i3} & = & [\begin{array}{cccccc} -\gamma I & 0 & 0 & 0 & \bar{\tau }P_{i} & 0\\ {}* & -\Lambda_{1i}+{\bar{d}}^{2}W & 0 & 0 & \bar{\tau }A_{i}^{T}P_{i} & 0\\ {}* & * & -\Lambda_{2i} & 0 & \bar{\tau }B_{i}^{T}P_{i} & 0\\ {}* & * & * & -W & \bar{\tau }D_{i}^{T}P_{i} & 0\\ {}* & * & * & * & R-2P_{i} & 0\\ {}* & * & * & * & * & R-2P_{i} \end{array}]. \end{array}$$ (66)

Then, it can be derived by Lemma 3 that $E\{LV\left(t\right)+\gamma^{-1}\theta e^{T}\left(s\right)e\left(s\right)-2\left(1-\theta \right)e^{T}\left(s\right)\varpi \left(s\right)-\gamma \varpi^{T}\left(s\right)\varpi \left(s\right)\}<0$ holds, if Π_*i*_ < 0. Therefore, under zero initial condition, it can be obtained by integrating both sides of Eq (48) that *J* ≤ 0 holds, which means that the mixed *H*_∞_ and passive synchronization of the Markov jump neural networks is well achieved according to Definition 2 and completes the proof.

**Theorem 2.** *For given upper bounds of mixed time-varying delays* $\bar{\tau }$ *and* $\bar{d}$, *and given scalars* *θ* *and* *γ*, *the mixed* *H*_∞_ *and passive synchronization of the Markov jump neural networks* Eqs (1) *and* (6) *can be achieved in the sense of Definition 1 and 2, if there exist mode-dependent symmetric matrices* *P*_*i*_ > 0, *mode-dependent matrices* *V*_*i*_, *symmetric matrices* *Q* > 0, *R* > 0 *and a constant* *ε* > 0 *such that*

$$\begin{array}{c} \Psi_{i}:=[\begin{array}{cc} \Psi_{i1} & \Psi_{i2}\\ {}* & \Psi_{i3} \end{array}]<0 \end{array}$$ (67)

*hold for all* i∈I, *respectively, where*

$$\begin{array}{ccc} \Psi_{i1} & := & [\begin{array}{cc} \Psi_{i11} & \Psi_{i12}\\ {}* & \Psi_{i13} \end{array}], \end{array}$$ (68)

$$\begin{array}{ccc} \Psi_{i11} & := & [\begin{array}{cc} \begin{array}{c} V_{i}+V_{i}^{T}-P_{i}C_{i}-C_{i}^{T}P_{i}^{T}+\sum_{j=1}^{N}\pi_{ij}P_{j}\\ +Q-R-\hat{F}\Lambda_{1i}+\gamma^{-1}\theta I \end{array} & R\\ {}* & -2R-\hat{F}\Lambda_{2i} \end{array}], \end{array}$$ (69)

$$\begin{array}{ccc} \Psi_{i12} & := & [\begin{array}{ccc} 0 & P_{i}-\left(1-\theta \right)I & P_{i}A_{i}+\overset{ˇ}{F}\Lambda_{1i}\\ R & 0 & \overset{ˇ}{F}\Lambda_{2i} \end{array}], \end{array}$$ (70)

$$\begin{array}{ccc} \Psi_{i13} & := & \text{diag}\{-Q-R,-\gamma I,-\Lambda_{1i}+{\bar{d}}^{2}W\}, \end{array}$$ (71)

$$\begin{array}{ccc} \Psi_{i2} & := & [\begin{array}{cccccc} P_{i}B_{i} & P_{i}D_{i} & \bar{\tau }V_{i}^{T}-\bar{\tau }C_{i}^{T}P_{i} & 0 & \delta P_{i}H_{i} & \varepsilon E_{i}^{T}\\ 0 & 0 & 0 & 0 & 0 & 0\\ 0 & 0 & 0 & 0 & 0 & 0\\ 0 & 0 & \bar{\tau }P_{i} & 0 & 0 & 0\\ 0 & 0 & \bar{\tau }A_{i}^{T}P_{i} & 0 & 0 & 0 \end{array}], \end{array}$$ (72)

$$\begin{array}{ccc} \Psi_{i3} & := & [\begin{array}{cccccc} -\Lambda_{2i} & 0 & \bar{\tau }B_{i}^{T}P_{i} & 0 & 0 & 0\\ {}* & -W & \bar{\tau }D_{i}^{T}P_{i} & 0 & 0 & 0\\ {}* & * & R-2P_{i} & 0 & \bar{\tau }\delta P_{i}H_{i} & 0\\ {}* & * & * & R-2P_{i} & \sqrt{\delta (1-\delta )}\bar{\tau }P_{i}C_{i}H_{i} & 0\\ {}* & * & * & * & -\varepsilon I & 0\\ {}* & * & * & * & * & -\varepsilon I \end{array}], \end{array}$$ (73)

$\hat{F}$, $\overset{ˇ}{F}$ *are defined in* Eq (22) and the controller gain can be obtained as $K_{i}=P_{i}^{-1}V_{i}$.

*proof.* Let *V*_*i*_ = *P*_*i*_ *K*_*i*_. The rest of the proof can follow from the proof of Theorem 1 directly.

## Discussion

In order to verify our designed synchronization scheme, the following simulation example is presented.

Consider the Markov jump neural networks with two modes, where

$$\begin{array}{ccc} C_{1} & = & [\begin{array}{cc} 2.1 & 0\\ 0 & 2 \end{array}],A_{1}=[\begin{array}{cc} 0.2 & 0.3\\ 0.4 & 0.1 \end{array}],B_{1}=[\begin{array}{cc} 0.3 & 0.5\\ 0.6 & 0.4 \end{array}],D_{1}=[\begin{array}{cc} 0.2 & -0.3\\ 0.3 & 1.1 \end{array}],\\ C_{2} & = & [\begin{array}{cc} 2.3 & 0\\ 0 & 2.4 \end{array}],A_{2}=[\begin{array}{cc} 0.3 & -0.2\\ 0.1 & 0.3 \end{array}],B_{2}=[\begin{array}{cc} 0.8 & 0.7\\ -0.5 & 0.2 \end{array}],D_{2}=[\begin{array}{cc} 0.4 & 0.1\\ -0.6 & 0.5 \end{array}], \end{array}$$

and the neuron activation function is taken as

$$\begin{array}{c} f_{1}\left(x\left(t\right)\right)=f_{2}\left(x\left(t\right)\right)=\tanh\left(x\left(t\right)\right), \end{array}$$

which satisfies Assumption 1 with $F_{1}^{-}=F_{2}^{-}=0$ and $F_{1}^{+}=F_{2}^{+}=1$, such that

$$\begin{array}{ccc} \hat{F} & = & \text{diag}\{0,0\},\\ \overset{ˇ}{F} & = & \text{diag}\{0.5,0.5\}. \end{array}$$

In the simulation, the transition probability matrix is given as

$$\begin{array}{c} \Theta =[\begin{array}{cc} -0.6 & 0.6\\ 0.4 & -0.4 \end{array}], \end{array}$$

where the time step is set as Δ*t* = 0.01.

The time-varying delays are assumed to be *τ*(*t*) = 0.25 + 0.05 sin *t* and *d*(*t*) = 0.15 + 0.05 cos *t*, such that $\bar{\tau }=0.3$ and $\bar{d}=0.2$. The disturbance ϖ(t) is supposed to be ϖ(t)=diag{0.05sint,0.05cost.} The parameters *δ*, *θ* and *γ* are set by *δ* = 0.5, *θ* = 0.4 and *γ* = 0.2.

The controller gain fluctuation satisfies the condition Eq (9) with

$$\begin{array}{ccc} H_{1} & = & \text{diag}\{0.1,0.1\},\\ {ϒ}_{1}\left(t\right) & = & \text{diag}\{\sin t,\cos t\},\\ E_{1} & = & \text{diag}\{0.5,0.5\},\\ H_{2} & = & \text{diag}\{0.2,0.2\},\\ {ϒ}_{2}\left(t\right) & = & \text{diag}\{\cos t,\sin t\},\\ E_{2} & = & \text{diag}\{0.4,0.4\}. \end{array}$$

By solving Ψ_*i*_ < 0, *i* = 1, 2 in Theorem 2, the mode-dependent controller gains can be obtained as follows:

$$\begin{array}{ccc} K_{1} & = & [\begin{array}{cc} -2.4702 & -0.6356\\ -0.6956 & -2.8230 \end{array}],\\ K_{2} & = & [\begin{array}{cc} -3.0688 & 0.2705\\ 0.0876 & -2.7356 \end{array}]. \end{array}$$

Set the initial values as *x*(*t*) = [1, −1]^*T*^ and *y*(*t*) = [−5, 5]^*T*^, respectively. Under the modes evolution shown in S1 Fig, it can be seen from S2 and S3 Figs that the synchronization can be achieved with the designed mode-dependent controllers, which demonstrates our control scheme.

## Conclusion

The non-fragile mixed *H*_∞_ and passive synchronization problem of Markov jump neural networks with mixed time-varying delays is addressed. By utilizing the stochastic stability theory, delay-dependent criteria are derived for ensuring that the desired synchronization is achieved and the non-fragile synchronization controller is designed. An interesting further research direction is extending the derived results to the uncertainty cases.

## Supporting information

S1 Fig The jumping modes of the neural networks. (TIF)

S2 Fig The controlled synchronization error of the neural networks. (TIF)

S3 Fig The control input of the neural networks. (TIF)
