# Supplementary figures and images for: Non-fragile mixed H∞ and passive synchronization of Markov jump neural networks with mixed time-varying delays and randomly occurring controller gain fluctuation
Source: PLoS One. 2017 Apr 14;12(4):e0175676. doi: 10.1371/journal.pone.0175676 (PMC5391947; doi:10.1371/journal.pone.0175676)

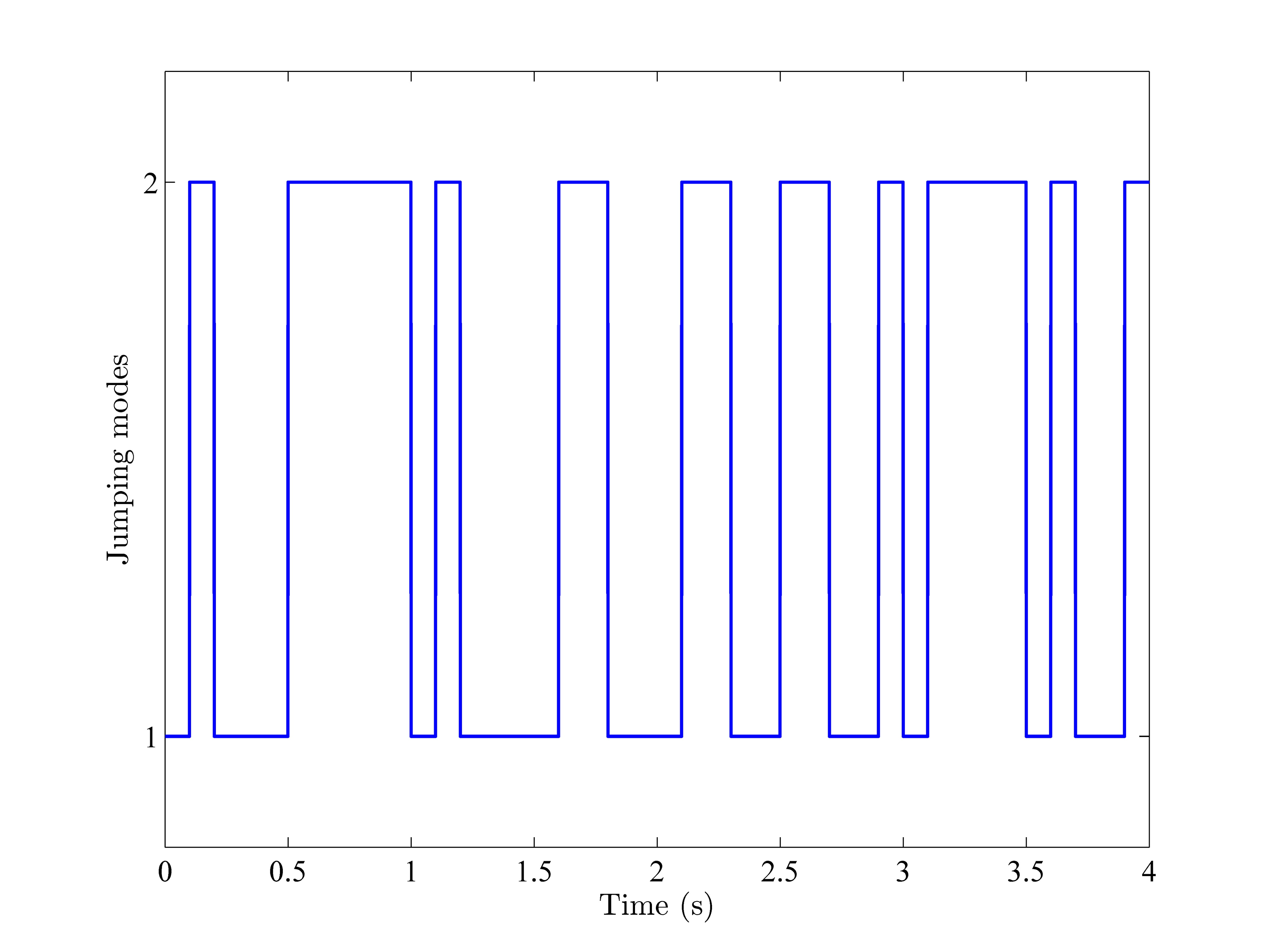

Supplement: S1 Fig — (TIF) [file pone.0175676.s001.tif]

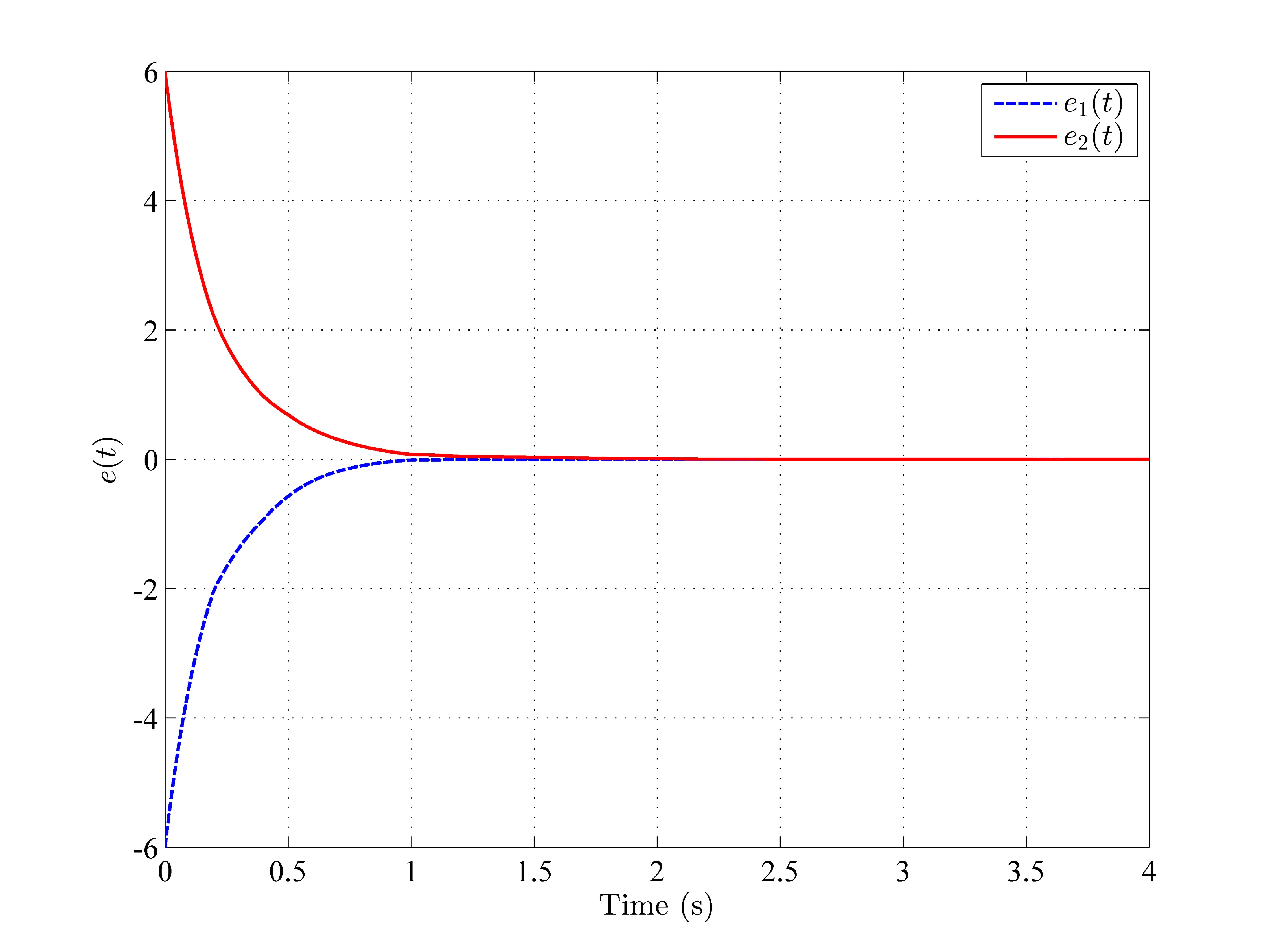

Supplement: S2 Fig — (TIF) [file pone.0175676.s002.tif]

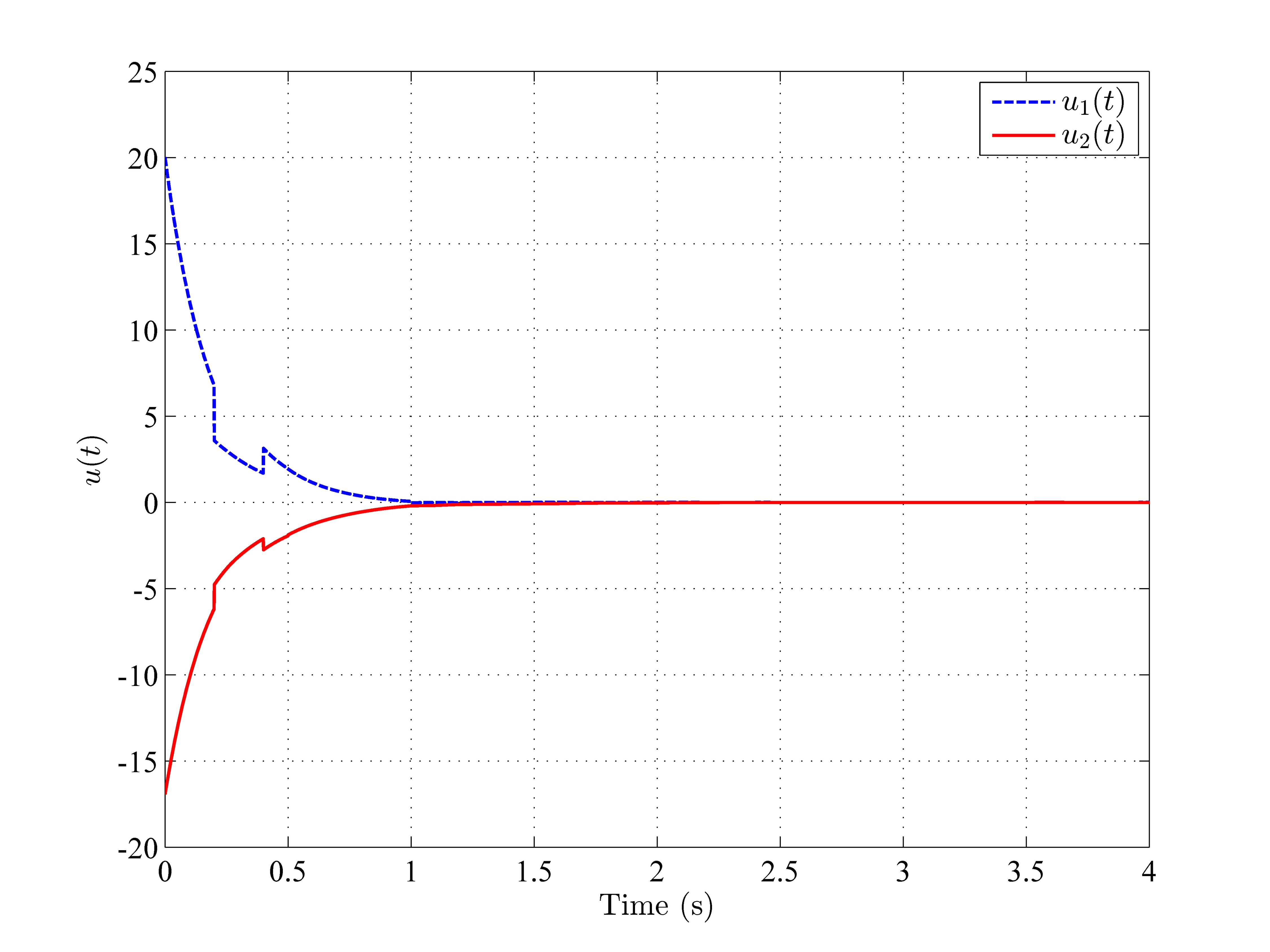

Supplement: S3 Fig — (TIF) [file pone.0175676.s003.tif]
